# Levels of the Mahogunin Ring Finger 1 E3 Ubiquitin Ligase Do Not Influence Prion Disease

**DOI:** 10.1371/journal.pone.0055575

**Published:** 2013-01-30

**Authors:** Derek Silvius, Rose Pitstick, Misol Ahn, Delisha Meishery, Abby Oehler, Gregory S. Barsh, Stephen J. DeArmond, George A. Carlson, Teresa M. Gunn

**Affiliations:** 1 McLaughlin Research Institute, Great Falls, Montana, United States of America; 2 Institute for Neurodegenerative Diseases and Department of Pathology, University of California San Francisco, San Francisco, California, United States of America; 3 Departments of Genetics and Pediatrics, Stanford University, Stanford, California, United States of America; Case Western Reserve University, United States of America

## Abstract

Prion diseases are rare but invariably fatal neurodegenerative disorders. They are associated with spongiform encephalopathy, a histopathology characterized by the presence of large, membrane-bound vacuolar structures in the neuropil of the brain. While the primary cause is recognized as conversion of the normal form of prion protein (PrP^C^) to a conformationally distinct, pathogenic form (PrP^Sc^), the cellular pathways and mechanisms that lead to spongiform change, neuronal dysfunction and death are not known. Mice lacking the Mahogunin Ring Finger 1 (MGRN1) E3 ubiquitin ligase develop spongiform encephalopathy by 9 months of age but do not become ill. In cell culture, PrP aberrantly present in the cytosol was reported to interact with and sequester MGRN1. This caused endo-lysosomal trafficking defects similar to those observed when *Mgrn1* expression is knocked down, implicating disrupted MGRN1-dependent trafficking in the pathogenesis of prion disease. As these defects were rescued by over-expression of MGRN1, we investigated whether reduced or elevated *Mgrn1* expression influences the onset, progression or pathology of disease in mice inoculated with PrP^Sc^. No differences were observed, indicating that disruption of MGRN1-dependent pathways does not play a significant role in the pathogenesis of transmissible spongiform encephalopathy.

## Introduction

Transmissible spongiform encephalopathies, are rare but invariably fatal neurodegenerative disorders that affect humans and animals [1], [2]. They are associated with misfolding and aggregation of the cellular prion protein, PrP^C^, into a protease-resistant, pathogenic conformer referred to as PrP^Sc^, with Sc referring to the prototypical Scrapie prion disease of sheep. PrP^Sc^ can be generated and propagated from endogenous PrP^C^ following infectious exposure to exogenous PrP^Sc^, while rare inherited forms, such as familial Cruetzfeldt-Jakob disease, fatal familial insomnia and Gerstmann-Sträussler-Scheinker syndrome, result from autosomal dominant mutations in the prion protein gene (*PRNP*). Most prion diseases are characterized by spongiform changes, starting with the development of vacuoles in the neuropil and progressing to widespread vacuolation of the central nervous system (CNS). At advanced stages, there is typically neuronal loss, astrogliosis and cerebellar atrophy (predominantly affecting granular neurons), but no inflammatory response. Despite progress in understanding the primary cause of prion diseases, the cellular and molecular mechanisms that lead to neurodegeneration and death are still under investigation.

Mice lacking the E3 ubiquitin ligase, mahogunin ring finger-1 (MGRN1) or the type I transmembrane protein, attractin (ATRN) develop age-dependent CNS vacuolation that is histologically similar to that associated with prion diseases, without the accumulation of protease-resistant PrP^Sc^ [3], [4]. The cellular role of ATRN remains unknown, although it has been shown to be required for membrane homeostasis [5]. The only ubiquitination target of MGRN1 identified to date is tumor susceptibility gene 101 (TSG101), a component of the endocytic trafficking machinery that sorts membrane proteins into multivesicular bodies [6], [7]. Loss of MGRN1-dependent ubiquitination disrupts endo-lysosomal trafficking, leading to accumulation of activated epidermal growth factor receptor (EGFR) and alterations in the morphology of early endosomes, late endosomes and lysosomes. PrP is normally secreted and tethered to the plasma membrane by a GPI anchor, but ER stress and some pathogenic mutations in *PRNP* can induce mislocalization of PrP to the cytosol and induce non-transmissible neurotoxicity [8]. A recent study demonstrated that cytosolically exposed forms of PrP can bind to and sequester MGRN1 in HeLa cells [9], resulting in similar abnormalities in endo-lysosomal trafficking to those observed in cells in which *Mgrn1* was knocked down by siRNA. Over-expressing MGRN1 rescued the trafficking defects. Reduced immunostaining for MGRN1 was observed in the brains of transgenic mice expressing a transmembrane form of PrP, along with an age-dependent increase in lysosome size/number (based on Cathepsin D staining) in Purkinje cells. These data suggested that disrupted MGRN1-dependent endo-lysosomal trafficking could be the cellular mechanism underlying spongiform neurodegeneration in prion diseses.

Cytosolically-exposed PrP has been proposed to contribute to the pathogenesis of inherited and transmissible spongiform encephalopathies [8]. Mutations in the hydrophobic domain of the prion gene that lead to increased production of a transmembrane form with its N-terminal domain exposed to the cytosol cause neurodegeneration with pathology reminiscent of prion disease in transgenic mice [10]. Similar transmembrane forms of prion protein have been detected in both genetic and transmitted prion diseases [10]–[12]. The relationship between cytosolic PrP and CNS vacuolation is unclear. We tested whether functional sequestration of MGRN1 by cytosolic PrP contributes to transmissible prion disease by inoculating mice expressing reduced or elevated levels of *Mgrn1* with Rocky Mountain Laboratory (RML) prions. No differences were observed in the onset, progression, or histopathology of disease. This indicates that altered MGRN1 function has little or no role in the pathogenesis of transmissible prion diseases and indirectly supports a role for plasma membrane PrP^Sc^.

## Materials and Methods

### Ethics statement

All animal procedures adhered to Association for Assessment and Accreditation of Laboratory Animal Care guidelines and were approved by the Institutional Animal Care and Use Committee of the McLaughlin Research Institute.

### Mice

*Mgrn1^md-nc^* (null) mutant mice and *Tg(Mgrn1^I^)C3Tmg* transgenic (hereafter referred to as *Tg+*) mice, which express wild-type *Mgrn1* isoform I from the human ß-actin promoter, were described previously [4], [13]. The *Tg(Mgrn1^I^)C3Tmg* transgenic line completely rescue all aspects of the *Mgrn1* null mutant phenotype, including spongiform degeneration of the CNS. *Mgrn1* null mutant (*Mgrn1^md−nc^*) mice are maintained by breeding heterozygotes with their homozygous mutant siblings. A wild-type control line was established by inbreeding +/+ animals that were generated by intercrossing *Mgrn1^md−nc/+^* mice; this line is re-generated from *Mgrn1* heterozygotes every 3 years. *Tg+*; *Mgrn1* null mutant mice were backcrossed to wild-type mice to generate *Tg+* and *Tg− Mgrn1^md−nc/+^* and wild-type (*Mgrn1^+/+^*) mice. Mice were genotyped for the *Mgrn1^md−nc^* mutation and the transgene as previously described [13].

### *Mgrn1* expression

*Mgrn1* expression in the brains of *Tg+* and *Tg−* mice that were homozygous wild-type at the *Mgrn1* locus or heterozygous for the null allele (*Mgrn1^md−nc/+^*) was determined by quantitative RT-PCR. *Tg+* mice were obligate heterozygotes for the *Tg(Mgrn1^I^)C3Tmg* transgene since they were generated by mating *Tg+*; *Mgrn1^md−nc/+^* animals to *Tg−*; *Mgrn1^+/+^* mice. Brain RNA was extracted from 3 mice of each genotype using TriPure reagent (Roche), followed by DNaseI treatment (Invitrogen) and cDNA synthesis (High Capacity cDNA Kit, Applied Biosystems, Foster City, CA) prior to amplification using SYBR green Brilliant II PCR master mix (Agilent) on a BioRad Opticon II. All samples were analyzed in triplicate on a single plate. Expression was normalized against *glucose phosphate isomerase (Gpi)* levels using the comparative Ct method to determine the relative quantification value [14]. *Mgrn1* primers: GATCTACGGCATCGAGAACAA and AGTGTGTCCCGCAGGTC. *Gpi* primers: CAACTGCTACGGCTGTGAGA and CTTTCCGTTGGACTCCATGT.

### Prion inoculations

All procedures involving animals adhered to Association for Assessment and Accreditation of Laboratory Animal Care guidelines and were reviewed and approved by the Institutional Animal Care and Use Committee of the McLaughlin Research Institute. Female mice of the following genotypes were inoculated intracerebrally at 36–60 days of age with RML prions using 10 µl of 10% brain homogenate from clinically ill mice: *Tg−*; *Mgrn1^md−nc/+^* (n = 4), *Tg−*; *Mgrn1^+/+^* (n = 7), *Tg+*; *Mgrn1^md−nc/+^* (n = 6) and *Tg+*; *Mgrn1^+/+^* (n = 8). An independent cohort of 4 female and 3 male *Mgrn1^md−nc/+^* and 1 female and 8 male *Mgrn1^md−nc/md−nc^* mutant mice were also inoculated with RML prions; in these studies, *Mgrn1^md−nc/+^* mice were used as controls since they are phenotypically normal and a coisogenic *Mgrn1^+/+^* line was not available at the time. Animals were monitored daily for general health status, while neurologic status was assessed three times per week. At the first appearance of symptoms, mice were weighed prior to each neurological exam. Animals were euthanized when they showed signs of progressive neurological dysfunction or had lost 20% of their initial weight. One sagittal half of each brain was fixed in 10% formalin, while the other half was immediately frozen and stored at −80°C until processed for immunoblotting (see below). One-way ANOVA was used to test whether there were any significant differences in average time to onset of paresis or survival following inoculation.

### Immunoblotting

Mouse brain hemispheres were homogenized in 1.0 ml EMBO buffer (50 mM Tris-HCl pH 7.4, 150 mM NaCl, 1 mM EDTA 1% Triton X) per 0.1 g of brain weight. Whole protein was quantified using bicinchoninic acid assay (Pierce). For PrP^Sc^ detection, samples were digested with proteinase K (1 µg/50 µg total protein) for 1 h at 37 C, followed by addition of PMSF (2 mM final concentration). Samples (20 µg of total protein) were subjected to electrophoresis through 10 or 14% Novex Tris-Glycine gels (Invitrogen) and transferred to PVDF membranes. Blots were blocked with 5% non-fat dry milk in Tris-buffered saline with Tween-20 (TBST) for 1 h, incubated with anti-prion antibody D18 (0.5ug/mL) overnight at 4 C, then washed and incubated at room temperature for 2 hrs with HRP-conjugated goat anti-human (Bio-Rad) secondary antibody (1∶5000 in 1% not-fat dry milk) before being developed using ECL Western Blotting Substrate (Thermo Scientific Pierce).

### Histology

Formalin-fixed samples were processed, paraffin-embedded, and sectioned at 8 µm thickness. Sections of septum, hippocampus/thalamus, midbrain and cerebellum/brainstem were stained with hematoxylin and eosin (H&E, Fisher Scientific SH26–500D and 245–658) or processed by immunohistochemistry for PrP (R2 monoclonal antibody, a gift from Dr. Stanley Prusiner) or the astrocyte marker, GFAP (Dako #Z0334). The secondary antibody for R2 was biotinylated goat anti-human Kappa chain (Vector Laboratories #BA–3060) and for GFAP was biotinylated goat anti-rabbit IgG (Vector Laboratories #BA–1000). The Peroxidase substrate DAB kit (Vector Laboratories #SK–4100) was used for color development. The percentage area occupied by vacuoles in a high power field was measured on H&E stained sections from 2 animals of each genotype. Each brain region was scored on one section for each animal. A score of 0 indicates no vacuoles were present. A score of up to 5 could reflect a small amount of real vacuolation or artifact and is considered to represent ‘no significant lesion’ (NSL). Scores of 5–19, 20–69 and ≥70 indicate mild, moderate and severe vacuolation, respectively [15].

## Results

One possible mechanism by which prion replication may cause disease is by inducing misdirection of PrP to the cytoplasm [11]. Since *Mgrn1* over-expression rescued the endo-lysosomal trafficking defects associated with the presence of cytosolically exposed forms of PrP *in vitro* [9], we set out to test whether *Mgrn1* levels influence PrP^Sc^-mediated prion disease *in vivo* by inoculating mice that express no *Mgrn1* and mice that over-express *Mgrn1* with RML prions. A *Mgrn1* isoform I transgene (*Tg(Mgrn1^I^)C3Tmg*) that rescues all aspects of the *Mgrn1^md−nc/md−nc^* phenotype, including CNS vacuolation, was previously shown to be expressed in the brain [13] but its expression level relative to endogenous *Mgrn1* was not assessed. Since an antibody that recognizes endogenous MGRN1 in mouse brain lysates is not available, we performed quantitative RT-PCR to assess *Mgrn1* expression in the brains of *Tg(Mgrn1^I^)C3Tmg* transgenic and non-transgenic wild-type (*Mgrn1^+/+^*) and *Mgrn1^md−nc/+^* mice. *Mgrn1* mRNA in the brain showed statistically significant differences consistent with genotype: expression in non-transgenic *Mgrn1^md−nc/+^* brains was significantly reduced relative to wild-type samples, transgenic (Tg+) *Mgrn1^md−nc/+^* and *Mgrn1^+/+^* mice had significantly higher levels (3-4-fold) than their non-transgenic counterparts, and *Tg+*; *Mgrn1^md−nc/+^* brains expressed similar levels to wild-type brains (Table 1).

**Table 1:** Brain *Mgrn1* expression.

Genotype	*Mgrn1* relative quantification value (range)	p value^a^
*Tg−*; *Mgrn1^md−nc/+^*	0.42 (0.22–0.79)	0.04
*Tg−*; *Mgrn1^+/+^*	1.00 (0.82–1. 21)	n/a
*Tg+*; *Mgrn1^md−nc/+^*	1.41 (0.76–2.61)^b^	0.21
*Tg+*; *Mgrn1^+/+^*	4.43 (1.59–12.32)	0.001

a Student’s t-test against *Tg−*; *Mgrn1^+/+^* value, p<0.05 significant.^ b^Student’s t-test against *Tg−*; *Mgrn1^mdnc/+^* yields p = 0.04.

**Figure 1:**
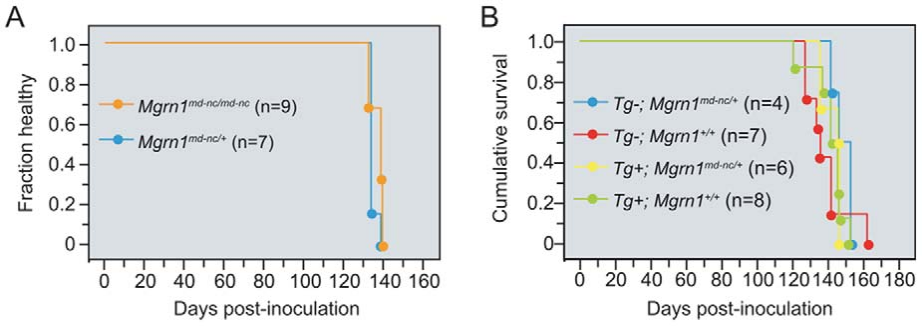
Kaplan-Meier plots for health status following RML prion inoculation. (A) Plot indicating proportion of healthy *Mgrn1^md−nc/+^* and *Mgrn1^md−nc/md−nc^* animals over time following RML prion inoculation. (B) Plot showing survival of *Mgrn1* transgenic (Tg+) and non-transgenic (Tg−) *Mgrn1^md−nc/+^* and *Mgrn1^+/+^* mice over time following inoculation with RML prions.

In our colony, *Mgrn1^md−nc^* null mutant mice start to show spongiform encephalopathy with reactive astrocytosis between 7 and 9 months of age. Unlike prion-inoculated mice, however, they do not develop obvious neurological symptoms and can live to at least 24 months of age. To test whether loss of MGRN1 function can contribute to the pathogenesis of prion disease, male and female mice homozygous for the *Mgrn1^md−nc^* null mutation and heterozygous controls were inoculated with RML prions and carefully monitored for signs of illness and neurological symptoms associated with prion infection (one or more of the following: weakness in rear, paresis, wobble in rear, abnormal gait, abnormal posture, generalized tremor, tail rigidity, poor righting reflex). No significant differences were observed in disease incubation time (Figure 1A). This indicates that absence of MGRN1 does not accelerate the pathogenesis of scrapie, but does not distinguish whether RML prions cause disease by disrupting MGRN1 function or act independent of MGRN1.

**Figure 2:**
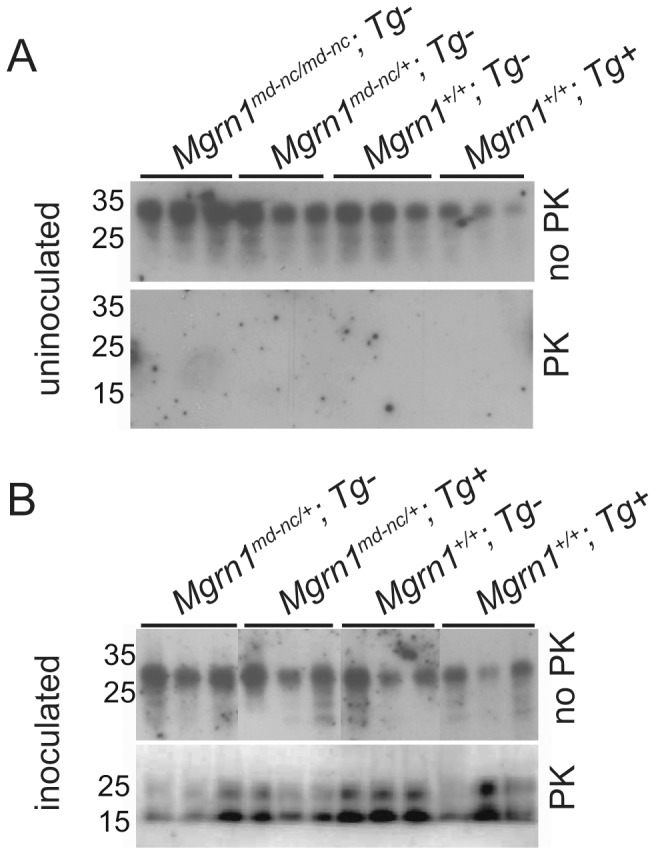
Brain PrP^C^ and PrP^Sc^ expression. (A-B) Brain protein lysates from uninoculated (A) and inoculated (B) transgenic and non-transgenic *Mgrn1^+/+^*, *Mgrn1^md−nc/+^* and/or *Mgrn1^md−nc/md−nc^* mice were subjected to immunoblotting with an antibody against PrP. The ‘no PK’ panel shows all PrP species present. The protease-resistant (proteinase K treated, ‘PK’) panel shows that PrP^Sc^ is present in samples from all RML prion-inoculated animals but not in uninoculated animals, regardless of *Mgrn1* genotype.

**Figure 3:**
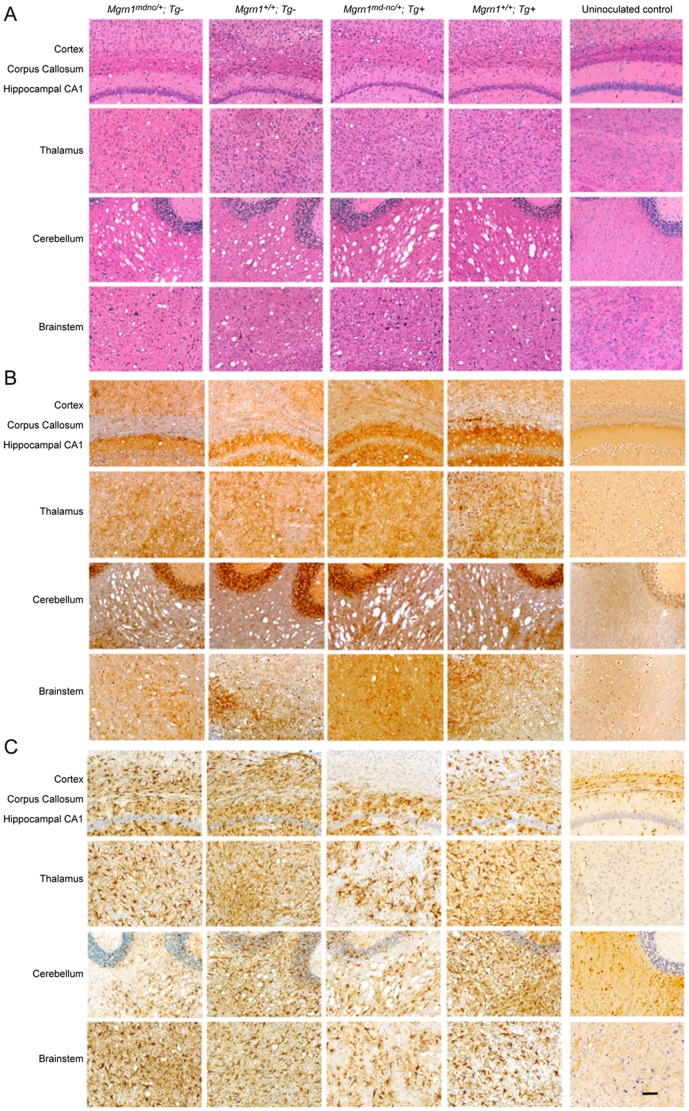
Histopathology and immunohistology of prion inoculated mice expressing normal or elevated levels of *Mgrn1*. (A) Hematoxylin and eosin-stained sections of indicated brain regions of non-transgenic and transgenic *Mgrn1^md−nc/+^* and *Mgrn1^+/+^* mice inoculated with RML prions and an uninoculated animal. Similar levels of vacuolation were observed in inoculated animals, regardless of genotype. As indicated in Table 2, the white matter of the cerebellum was most severely affected, followed by the brainstem and thalamus. (B) Immunohistochemistry against PrP on sections adjacent to those shown in A. The overall level and distribution of PrP was similar in inoculated mice regardless of their genotype. (C) Immunohistochemistry against GFAP on sections adjacent to those shown in A and B showing similar levels of astrocytosis in inoculated animals across genotypes. All images in–C were taken at the same magnification and are shown to same scale. Scale bar (in last panel): 100 µm.

As over-expression of MGRN1 in cell culture reversed the endosomal trafficking defects associated with the presence of cytosolic PrP, we tested whether *in vivo* over-expression of MGRN1 protected against RML prion-induced disease. Female non-transgenic and transgene-positive *Mgrn1^+/+^* and *Mgrn1^md−nc/+^* mice were inoculated with RML prions and carefully monitored for signs of illness and neurological symptoms. No statistically significant differences were observed in the average time to onset of symptoms or survival time between mice expressing different levels of *Mgrn1* (Table 2 and Figure 1B). Protease-resistant PrP was detected in brain lysates from inoculated mice of every genotype, but not in samples from uninoculated animals (Figure 2), indicating that *Mgrn1* levels do not affect the conversion of PrP^C^ to PrP^Sc^. Histological and immunohistological analyses revealed no obvious genotype-dependent differences in the distribution or degree of vacuolation (Figure 3A and Table 3), the level or distribution of PrP (using an antibody that recognizes PrP^C^ and PrP^Sc^) (Figure 3B), or in reactive astrocytic gliosis (using an antibody against GFAP) (Figure 3C) in the brains of inoculated animals. Vacuoles were most abundant in the white matter of the cerebellum, followed by the brainstem, but even in these regions it was only mild to moderate (Table 3). Interestingly, cerebellar vacuoles in *Mgrn1* null mutant mice are most common in the granule layer [13], which was the least vacuolated region of the cerebellum in all RML prion-inoculated animals in this study.

**Table 2:** Disease progression.

Genotype (n)	Average age at inoculation (range)	Average number of days post-inoculation to appearance of paresis (range) (SD)^a^	Average number of days post-inoculation to death or euthanasia (range) (SD)^a^
*Tg−*; *Mgrn1^md−nc/+^* (4)	53 (40–59)	103 (98–107) (6.4)	148 (141–152) (5.3)
*Tg−*; *Mgrn1^+/+^* (7)	47 (39–60)	103 (88–107) (11.6)	138 (127–160) (11.3)
*Tg+*; *Mgrn1^md−nc/+^* (6)	41 (36–60)	110 (94–115) (11.3)	142 (135–146) (5.6)
*Tg+*; *Mgrn1^+/+^* (8)	45 (37–59)	102 (94–111) (7.4)	141 (120–152) (9.5)

a No statistically significant differences by one-way ANOVA (all p>0.40).

**Table 3:** Severity of vacuolation.

	Average severity of vacuolation by brain region (scores)^a^										
Genotype	Ctx	Hipp	Thal	Hyp	Sep	Caud	Fbr	Mol	Gran	Wm	Bst
*Tg−*; *Mgrn1^md−nc/+^* (n = 2)	NSL (4, 5)	NSL (4, 0)	NSL (5, 4)	NS (0, 2)	NS (4, 2)	NSL (0, 1)	NSL (3, 0)	NSL (0, 0)	NSL (0, 4)	Mild (15, 10)	Mild (10, 7)
*Tg−*; *Mgrn1^+/+^* (n = 2)	NSL (0, 4)	NSL (3, 5)	Mild (6, 5)	NS (0,0)	NS (0, 4)	NSL (2, 0)	NSL (0, 0)	NSL (3, 0)	NSL (5, 0)	Mild (20, 15)	Mild (10, 10)
*Tg+*; *Mgrn1^md−nc/+^* (n = 2)	NSL (3, 0)	NSL (0, 3)	NSL (2, 5)	NS (2, 0)	NS (0, 0)	NSL (na, 0)	NSL (na, 0)	NSL (0, 0)	NSL (0, 0)	Mild (20, 30)	Mild (15, 10)
*Tg+*; *Mgrn1^+/+^* (n = 2)	NSL (0, 1)	NSL (3, 0)	NSL (3, 1)	NS (0, 0)	NS (0, 3)	NSL (0, 0)	NSL (0, 0)	NSL (0, 0)	NSL (3, 1)	Mild (30, 10)	Mild (8, 5)

a Actual vacuolation scores (percentage of area occupied by vacuoles) provided in parentheses. Brain regions: Ctx: cortex; Hipp: hippocampus; Thal: thalamus; Hyp: hypothalamus; Sep: septum; Caud: caudate nucleus; Fbr: basal forebrain; Mol: molecular layer of cerebellum; Gran: granule layer of cerebellum; Wm: white matter of cerebellum; Bst: brainstem. Severity scores: NSL (no significant lesions), average vacuolation score <5; Mild, average vacuolation score 5–19; Mod (moderate), average vacuolation score 20–69; na: brain region not examined.

## Discussion

*Mgrn1* null mutant mice develop CNS vacuolation that is similar both histologically and in its anatomical distribution to that observed in animals inoculated with RML prions. The possibility that the same pathogenic mechanism might cause spongiform change in *Mgrn1* null mutant mice and prion diseases was suggested by the observation that cytosolically exposed forms of PrP can bind to and sequester MGRN1 [9]. In that study, the presence of cytosolic PrP in HeLa cells caused abnormalities in endo-lysosomal trafficking similar to those observed when *Mgrn1* expression is knocked down by siRNA, and rescue of these defects by over-expression of MGRN1 was consistent with functional sequestration of MGRN1 by cytosolic PrP. Our results indicate that neither loss of *Mgrn1* nor its over-expression *in vivo* influences the onset, progression or outcome of disease caused by RML prion inoculation, including the distribution and severity of vacuolation. Since the *Tg(Mgrn1^I^)C3Tmg* transgene has been shown to rescue vacuolation associated with loss of *Mgrn1* [13], this suggests that loss of MGRN1-dependent cellular processes is not the underlying cause of spongiform encephalopathy caused by RML prions. Furthermore, our data suggest that either cytosolic PrP is not produced in this disease model or it does not play a significant role in the pathogenesis of transmissible prion diseases. We cannot, however, rule out the possibility that functional sequestration of MGRN1 may contribute to the neurotoxicity associated with cytosolic PrP.

Our results are consistent with other studies that have suggested cytosolic PrP does not make a significant contribution to prion disease, particularly the pathogenesis of CNS vacuolation. For example, transgenic mice expressing cytosolic PrP did not develop spongiform change, even when the transgene was expressed on a *Prnp* null background and the mice were inoculated with RML prions, nor did the inoculated mice get sick or accumulate protease-resistant PrP [16]. Spongiform change was not observed a patient carrying a truncation mutation in *PRNP* (Q160X) that has been shown to lead to significant cytoplasmic retention of PrP [17], [18]. In several studies, accumulation of cytosolic PrP was shown to not be toxic to human or mouse neurons in primary culture or to N2a cells [19]–[21], and in two of those studies, the presence of cytosolic PrP was in fact associated with protection against apoptosis. Together, these data suggest that the presence of cytosolic prion protein is not sufficient to cause prion disease and is not functionally equivalent to loss of MGRN1.

An alternative mechanism for vacuolar degeneration of neurons and their synapses is the accumulation of PrP^Sc^ in neuronal cellular membranes. At least 80% of PrP^Sc^ formed accumulates in the neuronal plasma membranes, especially in synaptic regions [22], and most vacuoles occur in pre- and post-synaptic structures [23]. In experimental scrapie and sporadic CJD, PrP^Sc^ accumulation and vacuolation begin focally in the brain and progress by axonal transport of PrP^Sc^ to different regions of the central nervous system [22]. The brain regions affected in the terminal stages of prion disease are determined by the strain of prions (PrP^Sc^) [24]. Neuronal dysfunction and morphological changes (vacuolation) appear to be caused directly by accumulation of PrP^Sc^ in plasma membranes [22], [25] and are related to the great effect of PrP^Sc^ has on membrane functions [26]–[28]. Dendritic degeneration, which is an additional abnormal step in synapse pathobiology, is caused specifically by PrP^Sc^ activation of Notch-1 signaling in the neuronal plasma cell membrane [29], [30]. Therefore the effects of PrP^Sc^ on membrane pathobiology cannot be ignored. PrP^Sc^ accumulates to a lesser degree by endocytosis into lysosomes and by phagocytosis into autophagosomes that release PrP^Sc^ into the extracellular space [25], and ingestion of PrP^Sc^ by activated microglia causes release of cytokines from microglia that cause nerve cell death [31].

The similar disease progression of *Mgrn1* null mutant mice, transgenic mice that over-express *Mgrn1*, and controls inoculated with RML prions indicates that MGRN1-dependent processes are not necessary for the pathogenesis of transmissible prion disease. Further studies, along with a better understanding of the origin of CNS vacuoles, will be needed to determine whether PrP^Sc^ and loss of MGRN1 act through the same downstream pathways to cause this intriguing phenotype.
